# Marketing and US Food and Drug Administration Clearance of Artificial Intelligence and Machine Learning Enabled Software in and as Medical Devices: A Systematic Review

**DOI:** 10.1001/jamanetworkopen.2023.21792

**Published:** 2023-07-05

**Authors:** Phoebe Clark, Jayne Kim, Yindalon Aphinyanaphongs

**Affiliations:** 1 Biomedical Informatics, NYU Langone Health, New York, New York; 2 New York University, New York, New York

## Abstract

**Importance:**

The marketing of health care devices enabled for use with artificial intelligence (AI) or machine learning (ML) is regulated in the US by the US Food and Drug Administration (FDA), which is responsible for approving and regulating medical devices. Currently, there are no uniform guidelines set by the FDA to regulate AI- or ML-enabled medical devices, and discrepancies between FDA-approved indications for use and device marketing require articulation.

**Objective:**

To explore any discrepancy between marketing and 510(k) clearance of AI- or ML-enabled medical devices.

**Evidence Review:**

This systematic review was a manually conducted survey of 510(k) approval summaries and accompanying marketing materials of devices approved between November 2021 and March 2022, conducted between March and November 2022, following the Preferred Reporting Items for Systematic Reviews and Meta-analyses (PRISMA) reporting guideline. Analysis focused on the prevalence of discrepancies between marketing and certification material for AI/ML enabled medical devices.

**Findings:**

A total of 119 FDA 510(k) clearance summaries were analyzed in tandem with their respective marketing materials. The devices were taxonomized into 3 individual categories of adherent, contentious, and discrepant devices. A total of 15 devices (12.61%) were considered discrepant, 8 devices (6.72%) were considered contentious, and 96 devices (84.03%) were consistent between marketing and FDA 510(k) clearance summaries. Most devices were from the radiological approval committees (75 devices [82.35%]), with 62 of these devices (82.67%) adherent, 3 (4.00%) contentious, and 10 (13.33%) discrepant; followed by the cardiovascular device approval committee (23 devices [19.33%]), with 19 of these devices (82.61%) considered adherent, 2 contentious (8.70%) and 2 discrepant (8.70%). The difference between these 3 categories in cardiovascular and radiological devices was statistically significant (*P* < .001).

**Conclusions and Relevance:**

In this systematic review, low adherence rates within committees were observed most often in committees with few AI- or ML-enabled devices. and discrepancies between clearance documentation and marketing material were present in one-fifth of devices surveyed.

## Key Points

**Question:**

Are medical devices that are marketed as enabled for artificial intelligence (AI) or machine learning (ML) being appropriately approved for AI or ML capabilities in their US Food and Drug Administration (FDA) 510(k) clearance?

**Findings:**

In this systematic review of 119 public 510(k) application summaries and corresponding marketing materials, devices with significant software components similar to devices flagged in the FDA’s published list of AI- or ML-enabled devices were defined and taxonomized into categories of adherent, contentious, and discrepant devices. Of 119 devices queried, 12.6% were considered discrepant, 6.7% were considered contentious, and 80.6% were consistent between marketing and FDA 510(k) clearance summaries.

**Meaning:**

These findings suggest that there were discrepancies between the marketing and 510(k) clearance of AI- or ML-enabled medical devices, with some devices marketed as having such capabilities not approved by the FDA for use of AI or ML.

## Teaser

This systematic review assesses US Food and Drug Administration (FDA) 501(k) clearance documents and marketing materials for adherence to guidelines for artificial intelligence– and machine learning–enabled medical devices.

## Introduction

Use of artificial intelligence (AI) in health care is widespread.^1,2,3^ Natural language processing^4^ techniques are used to understand clinical documentation and the implications of physician documentation. Convolutional neural networks help estimate molecule structure and determine effective pharmacological candidates for disease treatment. In the past decade, multiple machine learning (ML) techniques have been used to follow a similar pattern for image classification issues. In ML, scientists use supervised learning on existing labeled data to train a model and then evaluate its effectiveness in reader studies with human experts, with the eventual aim of these ML models being use as a diagnostic aid.

Medical devices must obtain US Food and Drug Administration (FDA) clearance or approval to be legally sold and used in the US. As devices are developed, approved, and marketed to consumers for use in health care settings, FDA approval provides reasonable assurance of legitimacy and safety to the consumer when considering using a device in such a sensitive field. Health care devices that use AI and ML must adhere to the same process, and once approved, the devices should be marketed accurately to inform consumers that their algorithms are safe and effective for public use.

### FDA Approvals

Guidelines and manuals for adherence and regulation of items approved for use are publicly available for all devices under FDA jurisdiction, except for most AI - or ML-enabled software and software as medical devices. In place of this is the Good Machine Learning Practice for Medical Device Development Guiding Principles,^5^ which outlines 10 guiding principles that, although generally adopted throughout all AI and ML developers, are not presented as staunch requirements to follow. Some are obvious, like the fact that training and test data should always remain separate. Others, such as “focus is placed on the performance of the human-AI team,” ask that “human interpretability of [model] outputs be addressed instead of just considering the model in isolation.”

The FDA has released an updated action plan regarding AI and ML technologies to notify the public and inform individuals and organizations potentially seeking device approvals of the FDA’s planned processes as well as what approvals might look like when the administration is fully equipped to handle these requests. The FDA intends to view ML technologies with a holistic approach, considering not only an item as presented at face value, but its ability to update, adapt, and shift in a short period of time. In January 2021,^6^ the FDA set out to build regulatory frameworks and solidify a “predetermined change control plan for software’s learning over time.” At the same time, a pamphlet^7^ was released that went into more depth on an action plan, as well as addressed common questions and concerns posed to the FDA by stakeholders in this particular field. A predetermined change control plan^8^ is addressed in this pamphlet, which includes a more realized Algorithm Change Protocol, through which the FDA hopes to find more transparency and real-world performance monitoring to evaluate any changes from premarket development through postmarket performance. Issues of bias in AI are prevalent in medical technologies, and this press release attempted to address that issue, calling for improved methods for bias elimination.

### Impact

The newer nature of AI as a part of the medical device industry in relation to regulatory bodies governing medical devices has led to a number of visible and potentially important issues in the approvals and postapprovals processes. The FDA’s function is to verify that there is a reasonable assurance that the devices are safe and effective for their intended uses, something consumers cannot verify on their own. Discrepancies between what consumers see in device marketing vs what the FDA considers safe are hard for consumers to reconcile. Clearance from the FDA implies endorsement of safety and effectiveness, and it is logical to assume potential consequences to this disconnect.

### Advisory Committee

The FDA uses several committees to receive advice on issues under their scope. The Medical Devices Advisory Committee^9^ is made up of 18 specialized panels, each of which is tasked with advising the commissioner on issues relating to their respective panels. Members of each individual panel are tasked with approving device applications that fall under the jurisdiction of their panel. As stated by the FDA, “The Center for Devices and Radiological Health has established advisory committees to provide independent, professional expertise and technical assistance on the development, safety and effectiveness, and regulation of medical devices and electronic products that produce radiation. Each committee consists of experts with recognized expertise and judgment in a specific field. Members have the training and experience necessary to evaluate information objectively and to interpret its significance.” Different advisory committees approve different numbers of devices and the frequency with which they encounter AI or ML-enabled devices is variable among committees. Since most devices approved by the FDA and flagged internally as AI or ML enabled fall under the jurisdiction of the radiology and cardiovascular committees, these committees are likely to be much more familiar with possible frameworks of devices enabled with AI or ML capabilities than, for example, the general and plastic surgery committee, which has reviewed fewer than 10 AI or ML devices.

### FDA Guidance Documentation

The FDA’s guidance document, “Guidance for the Content of Premarket Submissions for Software Contained in Medical Devices,”^10^ was issued in May 2005 and remained current as of 2018. The document is intended to guide developers when compiling documentation required in premarket submissions for software devices (devices that contain ≥1 software components, parts, or accessories, or are composed solely of software). An example of a software device might be the software used to operate a radiographic camera or a laser and could refer to the user interface of a device primarily used for its physical function or data-capturing abilities. The requirements on the part of submitting parties in this regard are mainly to determine the level of risk the device poses to the user (ie, *level of concern*). The extent of submission documentation that is recommended is then derived from the level of concern associated with the device. AI-enabled devices generally are of minor or moderate risk, assuming the suggestions of the device are further reviewed by a physician prior to acceptance. Consequently, the Software Requirements Specification, which documents the requirements for the software and is recommended to be included in the premarket submission, typically consists mainly of hardware requirements needed to run the device and may more broadly include internal software tests and checks.

In November 2021, the FDA distributed a draft guidance document to obtain comments on the outdated nature of the 2005 document.^11^ The new guidelines, if finalized and implemented by the FDA, recommend applicants submit evidence of the calculations used in analytical software. The draft guidance document also calls for concrete descriptions of the methods behind analysis and information regarding AI or ML algorithms used in the software.

The most recent update from the FDA in regards to AI- or ML-enabled devices is the release of the Technical Performance Assessment of Quantitative Imaging in Radiological Device Premarket Submissions,^12^ issued in June 2022. This guidance document is long awaited and much needed, with the prevalence of AI- and ML-enabled devices increasing steadily. This document only applies to quantitative imaging in radiological devices, although a significant number of principles can be broadly applied to AI- or ML-enabled devices in general.

## Methods

Using the Preferred Reporting Items for Systematic Reviews and Meta-analyses (PRISMA) reporting guideline, we manually reviewed all 510(k) applications cleared by the FDA from November 2021 to March 2022 (Figure). From these, 119 medical devices (eTable 1 in Supplement 1) with significant software components similar to devices flagged in the FDA’s published list of AI- or ML-enabled devices (eTable 2 in Supplement 1) were identified, regardless of whether or not they had already been flagged. We then defined and taxonomized individual categories of adherent, contentious, and discrepant devices. Adjusted percentage of adherence was calculated as (*adherent devices* [1] + *contentious devices* [0.5] + *discrepant devices* [0]) / *total devices*. Using this calculation, devices were sorted into their respective approval committees.

**Figure.**
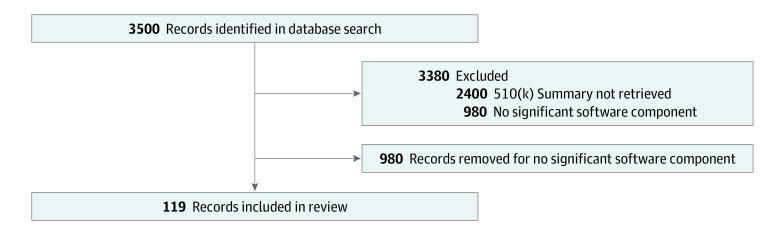
Flowchart of the Selected Materials

### Category Criteria

#### Adherent Devices

Adherent devices were categorized into 2 groups. The first was AI adherent devices, accompanied by summaries that mentioned at least once the presence of AI, ML applications, or proprietary algorithms used for diagnostic calculations. The second included queried devices that did not mention AI, ML applications, or proprietary algorithms in either the approval summary or in the marketing for the device but were investigated for the possibility of these capabilities. In both cases, the available marketing information for these devices echoed the sentiments of the FDA approval summary.

#### Contentious Devices

Contentious devices were devices that were not flagged by the FDA as AI or ML approved, nor did their 510(k) clearance summaries mention AI or ML. The marketing for these devices suggested AI or ML capabilities with the use of terms like *smart* and *predictive* analytics or modeling in reference to the device but did not outright state that the device was AI or ML enabled.

#### Discrepant Devices

The publicly available web pages for devices flagged as discrepant either stated they were enabled for AI or used AI-related language, like *machine learning*, *algorithm*, or *predictive analytics*. There was no mention in the FDA clearance summary of AI capabilities, and they were not listed in the FDA’s public list of AI- or ML-enabled devices.

### Statistical Analysis

A 1-way analysis of variance (ANOVA) test was performed using the Data Analysis tool of the Analysis ToolPak in Microsoft Excel version 2301. Statistical significance was set at *P* < .05. Data were analyzed from March to November 2022.

## Results

### Systematic Review

The systematic review of the data (Figure) generated 3500 screened devices and 1100 devices for which the 510(k) summary was available were sought for retrieval. The summaries were assessed for eligibility, and 119 devices with significant software components were included in our review.

### Statistical Analysis

A total of 96 devices (unadjusted, 80.67%; adjusted, 84.03%) were found to be adherent (44 adherent AI-enabled devices, 52 adherent non-AI–enabled devices). We found 8 devices (6.72%) that were contentious and 15 devices (12.61%) that were discrepant (Table). Most devices were from the radiological approval committees (75 devices [82.35%]), with 62 of these devices (82.67%) adherent, 3 (4.00%) contentious, and 10 (13.33%) discrepant; followed by the cardiovascular device approval committee (23 devices [19.33%]), with 19 of these devices (82.61%) considered adherent, 2 contentious (8.70%) and 2 discrepant (8.70%). ANOVA found that data from the cardiovascular and radiological devices advisory committee subsets were statistically significantly different among the 3 taxonomic categories (*P* < .001). Samples from the other advisory committees were not included in the ANOVA due to small sample sizes.

**Table.** Device Statistics Within Categories and by Advisory Committee

Advisory committee	Total devices, No. (column %)	Devices, No. (column %) [row %]^a^				Adherent devices, total No. (unadjusted %) [adjusted %]^b^
		Adherent		Contentious	Discrepant	
		AI	Without AI			
Radiology	75 (63.03)	38 (86.36) [50.67]	24 (46.15) [32.00]	3 (37.50) [4.00]	10 (66.67) [13.33]	62 (84.67) [82.67]
Anesthesiology	4 (3.36)	0	4 (7.69) [100]	0	0	4 (100) [100]
Clinical chemistry	6 (5.04)	1 (2.27) [16.67]	2 (3.85) [33.33]	2 (25.00) [33.33]	1 (6.67) [16.67]	3 (66.67) [50]
General and plastic surgery	1 (0.84)	0	0	0	1 (6.67) [100]	0
Cardiovascular	23 (19.33)	3 (6.82) [13.04]	16 (30.77) [69.57]	2 (25.00) [8.70]	2 (13.33) [8.70]	19 (86.96) [82.61]
Pathology	1 (0.84)	0	0	0	1 (6.67) [100]	0
Dental	3 (2.52)	0	2 (3.85) [66.67]	1 (12.50) [33.33]	0	2 (83.33) [66.67]
Orthopedic	1 (0.84)	0	1 (1.92) [100]	0	0	1 (100) [100]
Neurology	2 (1.68)	1 (2.27) [50.00]	1 (1.92) [50.00]	0	0	2 (100) [100]
Gastroenterology and urology	3 (2.52)	1 (2.27) [33.33]	2 (3.85) [66.67]	0	0	3 (100) [100]
Total	119 (100)	44 (36.97) [36.97]	52 (43.70) [43.70]	8 (6.72) [6.72]	15 (12.61) [12.61]	96 (84.03) [80.67]

Abbreviation: AI, artificial intelligence.

^a^ Column percentages indicate the proportion of devices that were adherent, contentious, or discrepant that fell within the jurisdiction of that advisory committee. Row percentages indicate the proportion of devices reviewed by that committee that were adherent, contentious, or discrepant.

^b^ Adjusted percentages were calculated as (*adherent devices* [1] + *contentious devices* [0.5] + *discrepant devices* [0]) / *total devices*).

### Example of an Adherent Device With AI

One example of an adherent device with AI was MammoScreen, with an FDA 510(k)^13^ summary that stated that “In MammoScreen, a range of medical image processing and machine learning techniques are implemented. The system includes ‘deep learning’ modules for recognition of suspicious calcifications and soft tissue lesions. These modules are trained with very large databases of biopsy-proven examples of breast cancer and normal tissue,” and it has been flagged in the FDA’s public list of AI- or ML-enabled devices. The marketing materials for MammoScreen also state plainly that the device is AI enabled.

### Example of Adherent Devices Without AI

An example of adherent device without AI was NightOwl, for which the 510(k) summary^14^ stated that it “is a wearable device intended for use in the recording, analysis, displaying, exporting, and storage of biophysical parameters to aid in the evaluation of sleep-related breathing disorders of adult patients suspected of sleep apnea.” It did not mention AI or ML.

After careful analysis of the marketing materials^15^ for this device, it is clear that the data were left to physicians for interpretation without intervention from any AI or ML; however, there was nothing beyond the FDA approval itself barring this device from generating prognostic analytics from the data collected.

### Example of a Contentious Device

An example of a contentious device was the Vector Computation ECG Mapping System. The FDA 510(k) summary^16^ describes that the device “leverage[s] analytical parameters from [an] externally developed models as part of the analysis to relate the input source signals to the final geometric output…which do not raise different questions of safety or effectiveness, as was further confirmed through the results of bench, usability, and clinical performance testing” The marketing for this device^17^ mentions the software combines proprietary computational modeling and that it is a smart device, but other information about the device requires requesting a pamphlet from the company.

### Example of Discrepant Devices

An example of a discrepant device is the NovaGuide 2 Intelligent Ultrasound. The FDA 510(k) summary^18^ states that this device “is a medical ultrasound system intended for use as an adjunct to standard clinical practices for measuring and displaying cerebral blood flow velocity and the occurrence of transient emboli within the bloodstream. The system assists the user in the setup and acquisition of cerebral blood flow velocity via the patient’s temporal windows.” While the extended portion of the summary mentions an algorithm used, it does not further discuss the AI capabilities. This device is not listed in the FDA’s public list of AI- or ML-enabled devices. The advertisement^19^ for this device states “With cutting-edge AI and advanced robotics, NovaGuide 2 uniquely captures blood flow data in real time to identify brain illnesses and diseases that present as changes in cerebral blood flow. NovaGuide 2 provides the clinical team with critical information to guide patient diagnosis and treatment.”

## Discussion

This systematic review found that the field of radiology had the most FDA-cleared devices using AI or ML. Device categories with fewer AI or ML device certifications and smaller device sample populations, like pathology and general and plastic surgery, may have had more difficulty reaching total adherence. A 0% adherence rate is likely not indicative of the oversight of a committee but instead showing the emergence of this type of device in that field. While radiological devices were strongly represented in clearances, they were still contributing discrepant devices to the market.

### Developer and Postmarket Monitoring Limitations

It is beyond the scope of this review to attempt to understand the reasoning behind this observation, but data exploration has provided the opportunity for conversation around how this issue persists. Developers of devices do not have a format to follow for 510(k) submission beyond the required components recommended by the FDA. Applicants can submit differently formatted applications, so a review of them does not produce a uniform schema of applications with software components. Mature companies with a history of FDA approvals likely have a more informed and standardized approach to submissions, while newer developers may not have the resources or knowledge to contribute 510(k) applications in the same way. Outdated guidance documentation for software used in medical devices is another likely contributing factor that has potential to be solved. Currently, developers must rely on outdated regulatory information when preparing a device submission until the newest draft document is finalized and able to supersede older guidance documentation. While the guidance document regarding technical performance assessment of quantitative imaging in radiological devices is comprehensive and has a strong foundation to generate trustworthy devices, it only applies to 1 type of device.

Another less evident factor in play may be the number of resources dedicated by the FDA to developments and advancements in AI and ML. The FDA oversees a huge amount of work in the US, and only approximately 10% of the agency’s resources are dedicated to devices and radiological health.^20^ Within that 10%, 35% of funding comes from industry user fees.^20^ Along with the FDA’s monetary contribution, this amounts to less than $630 000 for the budget of this branch as of 2021. Something so new and unregulated as AI cannot expect to receive most of this budget, considering everything else under the FDA’s jurisdiction, such as their recent role with helping mitigate the COVID-19 pandemic.^21,22^ To ask for marketing scrutiny on a new standard of devices among all of this is difficult.

### Limitations

This study has some limitations. Due to the inability to perform a keyword search on the FDA’s website containing 510(k) summaries, a manual review can only produce a sampling of devices. The marketing material for a number of devices was difficult or near impossible to obtain, and frequently marketing websites for devices required potential buyers to request a demonstration or consultation to receive further marketing material.

## Conclusions

This systematic review found that there was significant discrepancy in the marketing of AI- or ML-enabled medical devices compared with their FDA 510(k) summaries. Further qualitative analysis and investigation into these devices and their certification methods may shed more light on the subject, but any level of discrepancy is important to note for consumer safety. The aim of this study was not to suggest developers were creating and marketing unsafe or untrustworthy devices but to show the need for study on the topic and more uniform guidelines around marketing of software-heavy devices.

## Notes

Supplement 1. **eTable 1.** Queried Device Information and Corresponding Marketing Evidence, if Applicable **eTable 2.** Artificial Intelligence and Machine Learning (AI/ML)-Enabled Medical Devices

Supplement 2.
Data Sharing Statement
