# Supplementary material for: Marketing and US Food and Drug Administration Clearance of Artificial Intelligence and Machine Learning Enabled Software in and as Medical Devices: A Systematic Review
Source: JAMA Netw Open. 2023 Jul 5;6(7):e2321792. doi: 10.1001/jamanetworkopen.2023.21792 (PMC10323702; doi:10.1001/jamanetworkopen.2023.21792)
Supplement: Supplement 2. — Data Sharing Statement [file jamanetwopen-e2321792-s002.pdf]

# Data Sharing Statement

Clark. Marketing and US Food and Drug Administration Clearance of Artificial Intelligence and Machine Learning Enabled Software in and as Medical Devices. *JAMA Netw Open*. Published July 05, 2023. doi:10.1001/jamanetworkopen.2023.21792

**Data**
**Data available:** Yes
**Data types:** Other (please specify)
**Additional Information:** FDA Queried List of 510(k) Cleared Medical Devices and Marketing Information
**How to access data:** Uploaded PDF
**When available:** With publication

**Supporting Documents**
**Document types:** None

**Additional Information**
**Who can access the data:** N/a
**Types of analyses:** any purpose
**Mechanisms of data availability:** signed data access agreement
